# Assessment of the methylome and the cognition in urban dwellers

**DOI:** 10.1192/j.eurpsy.2024.1413

**Published:** 2024-08-27

**Authors:** E. Abdelmoula, N. Bouayed Abdelmoula, B. Abdelmoula

**Affiliations:** ^1^LR AMC, Ecole Doctorale Sciences et Ingénierie Architecturales (ED-SIA), Tunis; ^2^Genomics of Signalopathies at the service of Precision Medicine- LR23ES07, Medical University of Sfax, Sfax, Tunisia

## Abstract

**Introduction:**

The epigenome involving chemical modifications of DNA and chromatin that modulates gene expression in response to external and environmental conditions is characterized by great plasticity and reacts by epigenetic marks such as methylation signatures that can be inherited across generations.

**Objectives:**

Urban dwellers likely adapt to the level and growth of urbanization and resulting environmental changes through epigenetic changes. The aim of this study is to present what is currently known about the DNA methylome (the information of DNA methylation of all cytosines in a genome) and cognition when humans are exposed to changing urban environments.

**Methods:**

We conducted a comprehensive review of the scientific literature using PubMed database with the following keywords: DNA methylation, brain and urbanity.

**Results:**

Our search revealed a scarcity of scientific articles reporting methylome studies with assessment of correlations between methylome, cognitive status and urban environment. Among these papers, a Chinese study (2021) found a significant correlation between childhood urbanicity and better cognitive performance by measuring genome-wide methylation profile using more than 850,000 genome-wide CpG sites. In this study, the authors suggested that the impact of childhood urbanicity on cognition is partially mediated by the methylome and brain structure/function in humans whose childhood urbanicity differed. Other studies using other research approaches, suggested that the impact of living in an urban area is linked to better performance in terms of working memory, processing speed and verbal learning. We also found that the vast majority of studies investigating DNA methylation involved in rapid adaptation to new environments, including urban environments, focused on plant and animal species.

**Conclusions:**

The effects of urbanization on human beings are a topic of ongoing debate. Some studies suggest that urbanization can have beneficial effects on cognition, while others find that it can have harmful effects. Quantitative studies of methylation and the correlations between methylome, cognition, and urbanicity offer new opportunities to measure these effects and gain a better understanding of their mechanisms.

**Disclosure of Interest:**

None Declared

